# 6-O-angeloylplenolin inhibits anti-IgE-stimulated human mast cell activation via suppressing calcium influx and ERK phosphorylation

**DOI:** 10.22038/IJBMS.2022.64132.14120

**Published:** 2022-05

**Authors:** Yangyang Yu, Dongxu Lin, Zhenyu Liu, Ran Fang, Siman Zheng, Yongxian Cheng, Zhong Huang, Chun Wai Ng, Hang Yung Alaster Lau

**Affiliations:** 1 Shenzhen University Health Science Center, Shenzhen, China; 2 Department and Institute of Urology, Tongji Hospital, Tongji Medical College, Huazhong University of Science and Technology, Wuhan, China; 3 GI Division, Shenzhen University General Hospital, Shenzhen, China; 4 School of Biomedical Sciences, Faculty of Medicine, The Chinese University of Hong Kong, Hong Kong, China

**Keywords:** 6-O-angeloylplenolin, Allergy, Inflammation, Mast cell, SKP1 inhibitor

## Abstract

**Objective(s):**

Mast cells are important immune cells that primarily localize in the interface between the host and external environment, and protect us from pathogen infection. However, they are also involved in the pathology of allergic diseases such as asthma and atopic dermatitis. A novel S phase kinase-associated protein 1 (SKP1) inhibitor 6-O-angeloylplenolin (6-OAP), was studied with its potential ability to alleviate the anti-IgE-induced inflammatory responses of primary human cultured mast cells (HCMCs) and LAD2 cell line.

**Materials and Methods:**

We isolated the HCMCs from the buffy coat of voluntary blood donors. The effects of 6-OAP on mast cell activation were evaluated by measuring degranulation, cytokine release, migration, calcium influx, and ERK phosphorylation using spectro-fluorescence assay, multiplex cytometric bead assay/ELISA, migration assay, Fluo-4 calcium flux assay, and western blot, respectively.

**Results:**

It was found that 6-OAP exerted anti-inflammatory effects on human mast cells by dose-dependently suppressing the anti-IgE-mediated degranulation and release of cytokines such as proinflammatory cytokines (IL-8 and TNF-α), growth factors (GM-CSF, VEGF, and FGF), and chemokines (CCL2 and CCL3) in HCMC and LAD2 cells. It also suppressed the migration of immature HCMCs induced by CXCL12. Moreover, the process of calcium influx and ERK phosphorylation in activated HCMC cells were inhibited by 6-OAP administration.

**Conclusion:**

Our results showed that 6-OAP inhibited anti-IgE-induced inflammatory responses of human mast cells via suppressing calcium influx and ERK phosphorylation.

## Introduction

6-O-angeloylplenolin (6-OAP) is an inhibitor targeting S phase kinase-associated protein 1 (SKP1), causing dissociation of SKP1 from SKP2(1). SKP1 is one of the components of the SKP1-cullin-F-Box (SCF) ubiquitin ligase complex. There are about 70 F-box proteins, which account for the substrate specificity of SCF ligases, mediating ubiquitination of different proteins in mammals (2).

E3 Ubiquitin ligases were divided into 3 main classes which include Cullin-based E3s (e.g., SCF complex), HECT-based E3s (e.g., E6-AP) and RING-finger based E3s (e.g., Cbl). Ubiquitination is crucial for a variety of physiological processes, including differentiation, innate and adaptive immunity, and cell survival. Moreover, it also exerts great influence on inflammation (3). Recently, considerable progress has been made in elucidating the molecular action of ubiquitination in transduction signals. Studies have suggested that aberrant ubiquitination modification plays an important role in the pathogenesis of inflammation, through activation of NF-κB (4, 5).

Mast cells are immunological effector cells for both innate and adaptive immunity. Immature mast cells derived from hematopoietic progenitors migrate from bone marrow to the bloodstream and then reside in specific peripheral tissues such as mucosal surfaces, connective tissues, nerves, and skin, where they mature with the help of multiple factors (6). Generally, activation of mast cells leads to the release of chemical mediators, heparin-like molecules, growth factors, angiogenesis factors, and pro-inflammatory factors (7). But abnormal mast cell activation has long been recognized to be involved in different diseases such as asthma, eczema, allergic rhinitis, and cancer.

Whilst the relationship between ubiquitin-protein ligase and mast cell activation is rarely studied, there are still some clues to suggest a correlation between them. Overexpressing the ubiquitin E3 ligase c-Cbl inhibited the process of degranulation and cytokine production in activated rat mast cell line RBL-2H3(8). While the functions of ubiquitination in signaling pathways of inflammation have been shown to be important (9), the effects of different families of ubiquitin ligases on the activation of mast cells remain unknown. We thus studied the effects of 6-OAP on IgE-dependent activation of human mast cells. In this study, we employed primary human cultured mast cells (HCMCs) derived from human blood CD34^+^ progenitor cells and the mast cell line LAD2 (Laboratory of Allergic Disease 2 ) to investigate the effects of 6-OAP on degranulation, inflammatory mediators release, chemotaxis, and associated mechanisms of the activated mast cell.

## Materials and Methods

***Regents and chemicals***

6-OAP was isolated from the medicinal plant *Centipeda minima*, and its purity reached 99.5% (10). Iscove’s Modified Dulbecco’s Medium (IMDM), stem cell factor, IL-3, IL-6, and chemokine (C-X-C motif) ligand 12 (CXCL12) were purchased from Invitrogen (MD, USA). IL-4 and IL-9 were purchased from PeproTech (Rehovot, Israel). ERK, phos-ERK primary antibodies and anti-rabbit HRP-conjugated secondary antibody were purchased from Cell Signaling Technology (MA, USA). Human myeloma IgE was purchased from Merck (Darmstadt, Germany). Anti-human IgE was purchased from Sigma-Aldrich (MO, USA). ELISA kits for interleukin-8 (IL-8) and tumor necrosis factor-α (TNF-α) were purchased from BD Biosciences (NJ, USA). Multiplex cytokine array for determining granulocyte-macrophage colony-stimulating factor (GM-CSF), vascular endothelial growth factor (VEGF), fibroblast growth factor (FGF), CC chemokine ligand 2 (CCL2), and CC chemokine ligand 3 (CCL3) were purchased from ExcellBio (Shanghai, China).

***Primary cultured human mast cell (HCMC)***

This study protocol was reviewed and approved by the Shenzhen University Health Science Center Ethical Review Board (No. 2019021). All experiments followed the ethics procedures, and the involved volunteers have been informed of research purposes and potential risks. Primary HCMCs were cultured and validated under the method described in the report by Tam *et al*. (11). Briefly, CD34^+^ progenitor cells were separated from fresh human buffy coats of voluntary blood donors using the MACS system (Miltenyi Biotec) according to the manufacturer’s guidance and maintained in complete IMDM medium at a density of 5×10^5^ cells/ml. To guarantee the formation of mast cell phenotype, the cells were kept in hypoxic condition (5% O_2_ and 5% CO_2_) for 2 weeks and then kept in normoxic condition (21% O_2_ and 5% CO_2_) for 4 weeks. For the first 6 weeks, the cells were maintained in a medium with 200 ng/ml SCF and 100 ng/ml IL-6, while 1 ng/ml IL-3, 15 ng/ml IL-9, and 10ng/ml IL-4 were supplemented in week 1, week 2, and week 6, respectively. Subsequently, the cells were maintained in a medium with 100 ng/ml SCF and 50 ng/ml IL-6 for another 3 weeks and the mature HCMC phenotype was confirmed by the positive expression of mast cell tryptase and chymase (Figure S1). Before further experiments, mature HCMCs were sensitized with 0.5 μg/ml human myeloma IgE overnight.

***Laboratory of Allergic Disease 2 (LAD2) cell***

LAD2 cells were kindly provided by A. Kirshenbaum and D. Metcalfe (NIH, USA). Cells were maintained in a StemPro-34 medium supplemented with 10 ml/l StemPro nutrient supplement, 1:100 penicillin-streptomycin, 2 mmol/l (mM) L-glutamine, 100 ng/ml human stem cell factor, and 50 ng/ml IL-6 in an atmosphere containing 5% CO_2_ at 37 °C. The culture medium was replaced every 2 weeks and the cells were kept at a density of 10^5^ cells/ml. Cells were sensitized with 0.5 μg/ml human myeloma IgE overnight prior to further treatment.

***Cell viability assay***

Sensitized HCMCs and LAD2 cells were seeded in 96-well plates in triplicate at a density of 1 × 10^4 ^cells per well. The cells were treated with 100 μl complete mediums with different concentrations of 6-OAP (0, 1.25, 2.5, 5, 10 μM) and activated by 1 μg/ml anti-IgE antibody for 8 hr the next day. The mediums were replaced with a mixture of 100 μl fresh complete medium and 50 μl XTT labeling solution (Roche Diagnostic, MA, USA) and then incubated at 37 °C for 1 hr. The absorbance value was detected at a wavelength of 450 nm using a microplate reader (Thermo Fisher Scientific, MA, USA).

***Histamine release assay***

Sensitized HCMC (5 × 10^4^) and LAD2 were suspended in 400 μl full HEPES-buffered Tyrode solution. The cells were incubated with 6-OAP for 1 hr and then stimulated with 1 μg/ml anti-IgE at 37 °C in a water bath. After 30 min incubation, 600 μl ice-cold buffer was aliquoted into the suspension. The cell pellet and supernatant were separated by centrifugation. The histamine contents were determined by spectro-fluorescence assay and were corrected for spontaneous histamine release. The percentages of degranulation were calculated by the following formula: degranulation (%) = (histamine content of supernatant/total histamine content of supernatant and cell pellet) × 100%.

***Cytokine release assay***

Sensitized HCMC (5 × 10^4^) and LAD2 were suspended in a 100 μl culturing medium with 10% fetal bovine serum (FBS). The cells were incubated with 6-OAP for 1 hr and then activated by 1 μg/ml anti-IgE for 24 hr in a 37 °C incubator. The concentrations of pro-inflammatory cytokines (IL-8, TNF-α) in cell supernatants were determined using an ELISA kit, while the concentrations of growth factors (GM-CSF, VEGF, and FGF) and chemokines (CCL2 and CCL3) were measured by multiplex cytokine array using flow cytometry. The results were also corrected for spontaneous basal releases.

***Cell migration***

Since the circulating mast cell precursors are recruited to peripheral tissues, where they undergo a period of maturation to become effector mast cells, we thus investigated the effect of 6-OAP on migration of immature HCMCs instead of mature HCMCs. 8 × 10^4^ immature HCMCs (cultured for 4 weeks) were suspended in a 100 μl culturing medium with 10% FBS. Aliquoted cell suspensions were seeded in the upper chambers of transwell plates insert (8 μm pore size; Corning, NY, USA), and the lower chambers were filled with 600 μl culturing medium supplemented with 50 nM CXCL12 in the presence or absence of 10 μM 6-OAP. The migrated cells were counted by a hematocytometer after 4 hr incubation.

***Calcium influx assay***

Sensitized HCMCs (1×10^4^) were suspended in a 100 μl culturing medium with 10% FBS and treated with or without 10 μM 6-OAP. The transient changes in intracellular calcium concentrations were determined by capturing fluorescence images of the cells in a time-lapse sequence using an inverted fluorescent microscope. That is, the cells were activated by 1 μg/ml anti-IgE at second 60 and the images were captured every second. Captured images were quantified for fluorescence intensities and normalized to the cell counts.

***Western blot***

Sensitized HCMCs (5 × 10^5^) were suspended in a 1 ml culturing medium with 10% FBS and seeded in 12-well plates overnight. The cells were subsequently incubated with 10 μM 6-OAP for 2 hr and then activated by 1 μg/ml anti-IgE for 3 hr. The cell pellets were harvested and lysed in RIPA buffer. The BCA Protein Assay kit was applied to quantify the total protein concentrations of cell lysates. A total of 20 μg sample protein was loaded and separated in 10% SDS-PAGE gel, and then transferred to nitrocellulose membrane. The membranes were blocked by 5% skim milk in PBS and incubated with 1:1000 ERK, and phos-ERK antibodies overnight at 4 ^o^C. After washing thrice, the membranes were incubated with 1:3000 anti-rabbit HRP-conjugated secondary antibodies. The HRP signals were detected using enhanced chemiluminescence (ECL) substrate followed by film exposure.

***Statistical analysis***

The data were presented as mean (standard error of the mean, SEM). Student’s t-test or one-way ANOVA followed by Dunnett’s *post-hoc* test was employed for comparison between cells with or without 6-OAP treatment. A two-tailed *P*<0.05 was considered significant difference.

## Results

***Anti-IgE-activated degranulation and pro-inflammatory cytokine release were inhibited by 6-OAP in LAD2 and HCMC***

**Figure 1:**
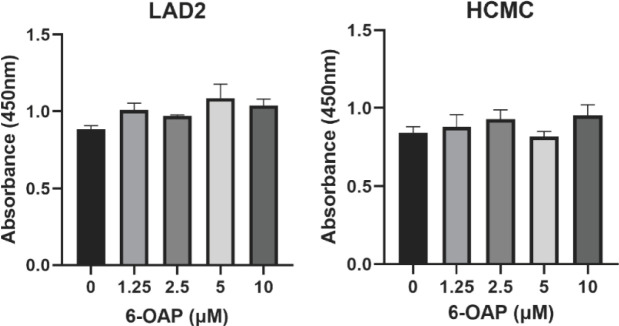
Effect of 6-OAP on cell viability of human mast cells human cultured mast cells (HCMC) and LAD2. No significant difference was observed in cell viability of mast cells after treatment with 6-O-angeloylplenolin (6-OAP)

Before evaluating the process of mast cell activation, the effect of 6-OAP on cytotoxicity was first determined. As shown in Figure 1, 6-OAP has no significant influence on cell viabilities of LAD2 and HCMC.

**Figure 2:**
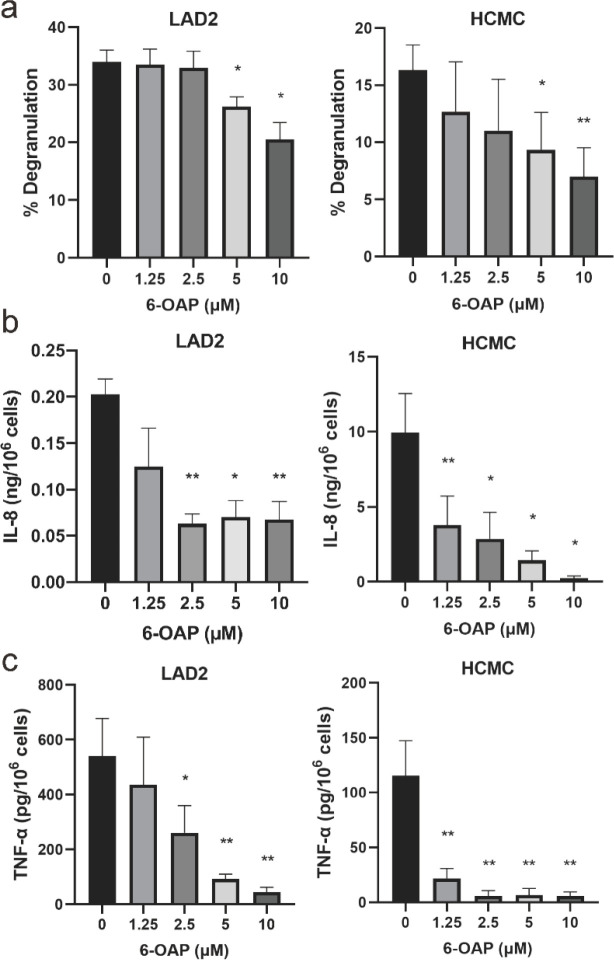
Histamine and pro-inflammatory cytokines released by human mast cells HCMC and LAD2 were inhibited by 6-O-angeloylplenolin (6-OAP). Sensitized mast cells were pre-incubated with 6-OAP for 8 hr and then stimulated with 1 µg/ml anti-IgE for promoting degranulation and cytokine release. The effects of various concentrations of 6-OAP on degranulation as indicated by histamine release (a), Interleukin-8 (IL-8) release (b), and Tumor necrosis factor-α (TNF-α) release (c) for LAD2 and human cultured mast cell (HCMC) are listed. All results were corrected for basal release. It is found that 6-OAP significantly suppressed the release of histamine, IL-8, and TNF-α in a dose-dependent manner in both LAD2 and HCMC cells. Data are presented as means ± SEM. *P<0.05 and **P<0.01 by one-way ANOVA

At the administrating concentration of 10 µM, 6-OAP suppressed the release of histamine from 34.00±2.04% to 20.50±2.97% in LAD2 cells, and from 16.33±2.19% to 7.00±2.52% in HCMC (Figure 2a). For IL-8 release of LAD2 and HCMC, both were inhibited dose-dependently by 10 µM 6-OAP from 0.215±0.017 ng/10^6^ to 0.075±0.017 ng/10^6^ cells, and from 9.96±2.60 ng/10^6^ cells to 0.236±0.162 ng/10^6^ cells, respectively (Figure 2b). Likewise, TNF-α released by LAD2 and HCMC were down-regulated continuously with increasing doses of 6-OAP. They were suppressed from 540.6±137.3 pg/10^6^ to 44.65±17.42 pg/10^6^ at 10 µM, and from 115.5±32.0 pg/10^6^ to about 5.97±4.82 pg/10^6^ at 2.5 µM then remained stable at low levels, respectively for LAD2 and HCMC (Figure 2c). 

All in all, 6-OAP suppressed degranulation and production of IL-8 and TNF-α dose-dependently in both anti-IgE-activated LAD2 and HCMC, suggesting that 6-OAP could inhibit allergic and pro-inflammatory responses of mast cells 

***Anti-IgE-activated growth factors and chemokine release were suppressed by 6-OAP in LAD2 and HCMC***

**Figure 3:**
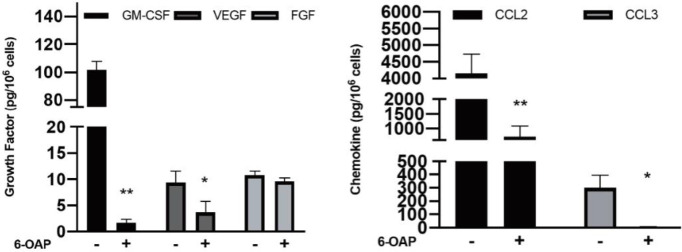
6-O-angeloylplenolin (6-OAP) regulated the release of growth factors and chemokines from anti-IgE-activated human cultured mast cell (HCMC). 5× 10^4^ sensitized HCMCs were pre-incubated with 10 µM 6-OAP for 8 hr and then stimulated with 1 µg/ml anti-IgE for 24 hr for cytokine release assay. The concentrations of cytokines were then determined with a multiplex cytometric bead array using flow cytometry, which was corrected by basal release. The production of growth factors (Granulocyte-macrophage colony-stimulating factor (GM-CSF) and Vascular endothelial growth factor (VEGF)) and chemokines (CCL_2_ and CCL_3_) induced by anti-IgE incubation were dramatically decreased in the presence of 6-OAP. Data are presented as means ± SEM. * *P*<0.05 and ** *P*<0.01 by student’s t-test

After examining the effect of 6-OAP on degranulation and pro-inflammatory cytokine release, the effects of 6-OAP on growth factors and chemokine release of HCMC and LAD2 cells were determined (Figure 3). 

The release of anti-IgE-induced GM-CSF, VEGF, CCL2, and CCL3 were down-regulated significantly by 10 µM 6-OAP from 101.95±5.85 pg/10^6^ cell to 1.74±0.57 pg/10^6^ cell, 9.41±0.80 pg/10^6^ cell to 3.65±2.13 pg/10^6^ cell, 4.15±0.58 ng/10^6^ cell to 0.72±0.37 ng/10^6^ cell, and 300.55±83.64 pg/10^6^ cell to 6.35±2.22 pg/10^6^ cell, respectively, while no significant difference was observed in FGF secretion. 

***Process of CXCL12-initiated immature HCMC migration was blocked by 6-OAP***

**Figure 4:**
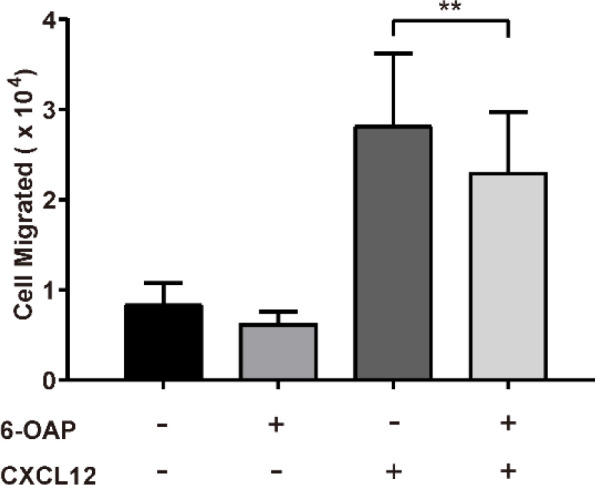
Effect of 6-O-angeloylplenolin (6-OAP) on migration of immature human cultured mast cell (HCMCs) induced by Chemokine (C-X-C motif) ligand 12 (CXCL12). Immature HCMCs were induced to migrate through an 8 μm transwell by 50 nM CXCL12 with or without the presence of 10 μM 6-OAP. The cells in the lower chamber were then counted and shown above. The immature HCMCs migrated into the lower chamber in response to CXCL12. However, the migration process was inhibited in the presence of 6-OAP. Data are presented as means ± SEM. * *P*<0.05 and ** *P*<0.01 by student’s t-test

The effect of 6-OAP on migration of immature mast cells (week 4 HCMC culture) was also examined (Figure 4). We can see that only (0.84±0.24) × 10^4^ immature mast cells migrated into the lower chamber without additional stimulation. In the presence of 50 nM CXCL12, (2.82±0.80) × 10^4^ cells migrated into the lower chamber. When 10 µM 6-OAP was added, the cell number of immature HCMC migration induced by CXCL12 was significantly reduced to (2.29±0.67) × 10^4^.

***Calcium mobilization provoked by anti-IgE stimulation was prohibited by 6-OAP in HCMC***

**Figure 5:**
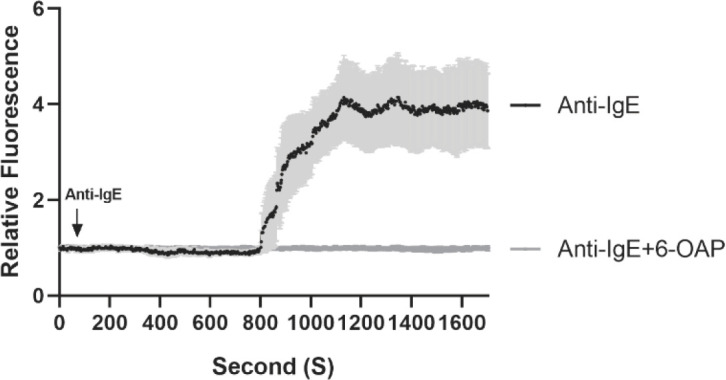
6-O-angeloylplenolin (6-OAP) inhibited calcium mobilization in anti-IgE-stimulated human cultured mast cell (HCMCs). Cells were preloaded with 1 µM Fluo-4-AM for 30 min at room temperature after 6-OAP pre-incubation (10 µM, 8 hr). Fluorescence images of the cultured cells were then captured under a fluorescence microscope for 28 min. It is revealed that 6-OAP blocked the process of calcium mobilization initiated by anti-IgE

Calcium mobilization is one of the crucial pathways of mast cell activation. Therefore, we sought to identify whether 6-OAP has an effect on calcium influx in activated HCMCs. HCMCs were pre-loaded with calcium dye Fluo-4-AM for 30 min in culture mediums. The dye was removed and the cells were resuspended in a culture medium and placed under a fluorescent microscope. HCMCs were activated by anti-IgE at second 60 after starting image recording. We found that the calcium signal dramatically increased at about second 800 as indicated by the increase of fluorescent intensity. However, in the presence of 6-OAP, the anti-IgE-induced calcium influx was abrogated (Figure 5).

***Inhibitory effects of 6-OAP on ERK phosphorylation in anti-IgE-activated HCMC***

**Figure 6:**
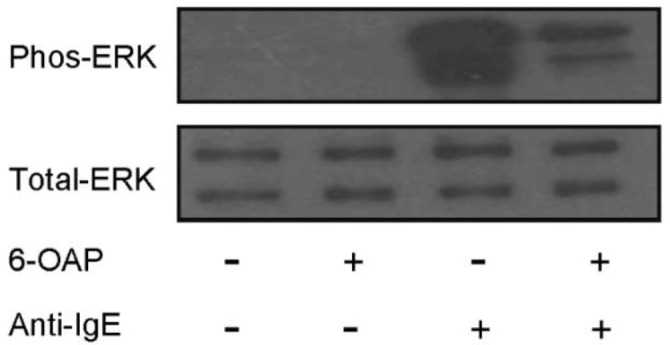
6-O-angeloylplenolin (6-OAP) inhibited ERK phosphorylation in anti-IgE-stimulated HCMC. Sensitized HCMCs were activated by 1 μg/ml of anti-IgE for 3 hr with or without 6-OAP pre-incubation (10 μM, 8 hr). The total proteins were harvested and separated by the western blotting method. As a result, 6-OAP prevented anti-IgE-induced ERK phosphorylation in HCMCs

The pathways that were affected by 6-OAP were further examined by determining ERK signaling (Figure 6). We can see that in the presence of 6-OAP, the phosphorylation of ERK induced by anti-IgE was inhibited.

## Discussion

Ubiquitination has been discovered as a common post-translational modification method and is involved in many cellular processes, such as protein degradation, gene expression, and cell activation; and its role in mast cell activation has also been reported in several literature sources. On one hand, E3 ubiquitin ligase CRL4A (CRBN) promoted proteasomal degradation of AMPK via ubiquitination, while inhibition of CRL4A (CRBN) stabilized AMPK activity but inhibited MAPK activity to reduce the release of allergic mediators in primary mouse bone marrow-derived mast cells (12). On the other hand, ubiquitin ligase c-Cbl negatively regulated the Src family kinase, Lyn, thereby inhibiting FcɛRI-mediated degranulation and cytokine production in the rat mast cell line RBL-2H3(8). Furthermore, Gal3, a positive regulator of FcɛRI ubiquitination, promoted cell adhesion and motility but inhibited antigen-mediated chemotaxis(13). In the current study, a novel identified Skp1 inhibitor, 6-OAP, had performed preferable anti-allergic effects via inhibiting degranulation, immunological mediator production, and chemotaxis, which was likely attributable to the blockade of calcium mobilization and ERK phosphorylation. 6-OAP is considered an inhibitor of SKP1 protein, an important component of the SCF ubiquitin ligase complex. SKP1 is required to stabilize F-box proteins in the SCF complex. Dissociation of SKP1 from NIPA, β-transducin repeats-containing proteins, and SKP2 lead to their degradation (14). 6-OAP was also responsible for SKP2 inhibition by inhibiting STAT3 transcriptional activity, so it exerted anti-tumor activity in lung cancer cells (15). The knowledge of the suppressed expression of those cytokines means that SKP1 plays an important role in antigen-induced activation of FcɛRI. It has been reported that E3 ubiquitin ligases could facilitate inflammatory responses (16, 17). In other words, inhibition of SKP1 by 6-OAP could be an option in mast cell-related hypersensitivity diseases.

Residing in the interfaces between the environment and our body, mast cells act as the first responder for the threats outside our body, and secrete lots of inflammatory mediators, which have been linked to inflammation and allergy. In response to antigen stimulation, mast cells rapidly discharge performed granule-stored mediators such as histamine, tryptase, and neutral proteases, followed by an acute-to-chronic inflammatory phase with production of *de novo* synthesized inflammatory mediators such as TNF-α and IL-8 (18, 19). Once the inflammatory cascade cannot be suppressed, the excessive generation of inflammatory mediators may cause persistent inflammatory damage. Our study highlighted the role of ubiquitination in the regulation of mast cell maturation and inflammatory responses and discovered that 6-OAP might serve as a novel compound to effectively treat mast cell hyperactivity-related diseases.

In response to external stimuli, the mast cells will undergo a complicated process, such as activation of the signaling cascade of Syk, calcium mobilization, or ERK pathway, to initiate an inflammatory response (20, 21). However, the function of ubiquitination in the process of mast cell activation was not well-studied. In the current study, mature HCMC treated with or without 6-OAP was subjected to RNA-seq analysis. GO and KEGG pathway enrichment analyses were performed to analyze the features of SCF complex-related genes in the differentially expressed gene sets (Table S1, Figure S2)*.* We found that 6-OAP could up-regulate the gene expression of SCF complex-related genes in HCMC, especially for genes associated with ubiquitin ligases, indicating that the inhibitory effect of 6-OAP in mast cell activation might be exerted by binding to ubiquitin ligases. Furthermore, we have demonstrated that calcium influx and phosphorylation of ERK were inhibited by 6-OAP in anti-IgE-activated human mast cells. Such results suggest that influx of calcium, which is important for inflammatory responses of human mast cells, is partly dependent on ubiquitination activation. A variety of immunological pathways were modulated by ubiquitination (22). As a matter of fact, ERK phosphorylation was associated with intracellular calcium influx. Therefore, it was no surprise that ERK phosphorylation was inhibited after reduction of calcium influx in the presence of 6-OAP. These data demonstrated that ubiquitination signaling played a crucial role in the early phase of mast cell activation after FcɛRI aggregation.

## Conclusion

SKP1 is a molecule that is crucial in signal transduction after FcɛRI activation. The SKP1 inhibitor 6-OAP exerted anti-inflammatory effects on anti-IgE-activated mast cells by blocking the essential calcium influx and regulating ERK phosphorylation, thus inhibiting degranulation and cytokine production. These data demonstrated that 6-OAP might be an alternative agent for treating mast cell-dependent type I hypersensitivity diseases. However, the exact target of 6-OAP would need to be further studied.

## Authors’ Contributions

YY and DL Contributed equally to this work as co-first authors; YY and DL Designed the experiments; DL, RF, and SZ Performed experiments and collected data; ZL and ZH Discussed the results and strategy; YY, YC, and CWN Supervised, directed, and managed the study; YY, DL, and HYAL Approved the version to be published.  

## Funding

This work was supported by the Research Grants of Shenzhen Science and Technology Plan Foundation Project (JCYJ20190808171601635,JCYJ20220518115604002), SZU Top Ranking Project (86000000210), and the National Natural Science Foundation of China (81800586).

## Conflicts of Interest

The authors have no conflicts of interest to declare.
